# Riddle of the Sphinx: Emerging Role of Transfer RNAs in Human Cancer

**DOI:** 10.3389/fphar.2021.794986

**Published:** 2021-12-15

**Authors:** Zhilin Qiu, Qin Wang, Lei Liu, Guozheng Li, Yi Hao, Shipeng Ning, Lei Zhang, Xin Zhang, Yihai Chen, Jiale Wu, Xinheng Wang, Shuai Yang, Yaoxin Lin, Shouping Xu

**Affiliations:** ^1^ Department of Breast Surgery, Harbin Medical University Cancer Hospital, Harbin, China; ^2^ CAS Center for Excellence in Nanoscience, CAS Key Laboratory for Biomedical Effects of Nanomaterials and Nanosafety, National Center for Nanoscience and Technology, Beijing, China

**Keywords:** transfer RNA, human cancer, biomarker, tumorigenesis, therapy

## Abstract

The dysregulation of transfer RNA (tRNA) expression contributes to the diversity of proteomics, heterogeneity of cell populations, and instability of the genome, which may be related to human cancer susceptibility. However, the relationship between tRNA dysregulation and cancer susceptibility remains elusive because the landscape of cancer-associated tRNAs has not been portrayed yet. Furthermore, the molecular mechanisms of tRNAs involved in tumorigenesis and cancer progression have not been systematically understood. In this review, we detail current knowledge of cancer-related tRNAs and comprehensively summarize the basic characteristics and functions of these tRNAs, with a special focus on their role and involvement in human cancer. This review bridges the gap between tRNAs and cancer and broadens our understanding of their relationship, thus providing new insights and strategies to improve the potential clinical applications of tRNAs for cancer diagnosis and therapy.

## Introduction

Cancer is one of the most complex diseases caused by multiple genetic disorders and cellular abnormalities. Its development and progression are regulated by multiple pathological processes (Bhawe and Roy, 2018), including environmental (Pan et al., 2018; Lewandowska et al., 2019), gender (Jara-Palomares et al., 2018), cultural (Tejeda et al., 2017; Lee, 2018), and lifestyle factors (Kerr et al., 2017; Jara-Palomares et al., 2018), as well as genetic mutations (Pak et al., 2015; Tejeda et al., 2017; Liew et al., 2019), epigenetic changes (Zhang and Huang, 2017; Porcellini et al., 2018; Liew et al., 2019), and abnormal signal transduction (Farooqi et al., 2018). Tremendous efforts have been made over the past decades with the aim of searching novel and more efficient tools in cancer therapy (Winn et al., 2016). However, cancer incidence and mortality remain high. Therefore, there is an urgent need to develop new strategies for early identification and more accurate diagnosis of cancer biomarkers for disease.

Recent studies on tRNAs have revealed the unexpected complexity of their structure and function (Schwartz et al., 2018). Besides participating in transcription and translation, tRNAs are also involved adaptive protein synthesis and can function as non-coding RNAs involving multiple regulatory networks (Schimmel, 2018). Moreover, some small RNAs previously thought as miRNAs were actually tRNA-derived small RNA (tsRNAs). Compared with miRNAs, tRNAs, tsRNAs, and tRNA-derived fragments (tRFs) are more stable and richer in biological fluids in solid cancers and blood malignant tumors (Zhang et al., 2009; Li et al., 2012). High expression of plasma exosome tRNAs in patients with lung cancer (Balatti et al., 2017), chronic lymphocytic leukemia (Veneziano et al., 2019), and liver cancer (Zhu et al., 2019) indicate that plasma exosome tRNAs may be involved in cancer development. These findings provide evidence that tRNAs and their derivatives may be potential diagnostic and therapeutic molecular biomarkers of cancer (Schageman et al., 2013; Dhahbi et al., 2014). In addition, mutation of the tRNA itself as well as supplementary proteins produced and modified by tRNA physiologically, are associated with cancer (Suzuki et al., 2011; Yao and Fox, 2013; Blanco and Frye, 2014). However, the exact molecular mechanisms by which tRNAs and tsRNAs are involved in cancer are unclear.

Moreover, in some cancers, the dysregulation of tRNAs can trigger the progression and proliferation of cancer cells by regulating transcription, translation, ribosome biogenesis and functioning as novel epigenetic factors. Although tRNAs and tsRNAs have been receiving increasing research attention, to date, there is no comprehensive summary of these findings, which would greatly be beneficial to future studies exploring tRNAs.

Herein, we detailed the current literature on tRNAs related to cancer. We comprehensively summarize the basic characteristics and functions of tRNAs, focusing on their involvement in various human cancers, particularly breast cancer, lung cancer, and melanoma. This review closes the gap between tRNAs and cancers and deepens our understanding of them, thereby providing new insights and strategies to guide researchers in further exploring the potential clinical applications of tRNAs in cancer diagnosis and treatment.

## Structure and Biogenesis of tRNAs and Their Derivatives

tRNAs are fundamental biological molecules that complete the flow of genetic information from DNA to protein by reading the cognate codons in the mRNA (Kirchner and Ignatova, 2015). Mature tRNAs in human cells are derived from precursor tRNAs (pre-tRNAs) containing 5′ leader and 3′ trailer sequences and introns in the anticodon loop (Phizicky and Hopper, 2010; Raina and Ibba, 2014). Mechanistically, pre-tRNAs are processed co- and post-transcriptionally to acquire their mature 5′ and 3′ ends, modified nucleosides, and the cloverleaf secondary structure, which contains four domains organized in unpaired and paired regions namely, acceptor arm, D arm, anticodon arm, and TΨC arm.

**FIGURE 1:**
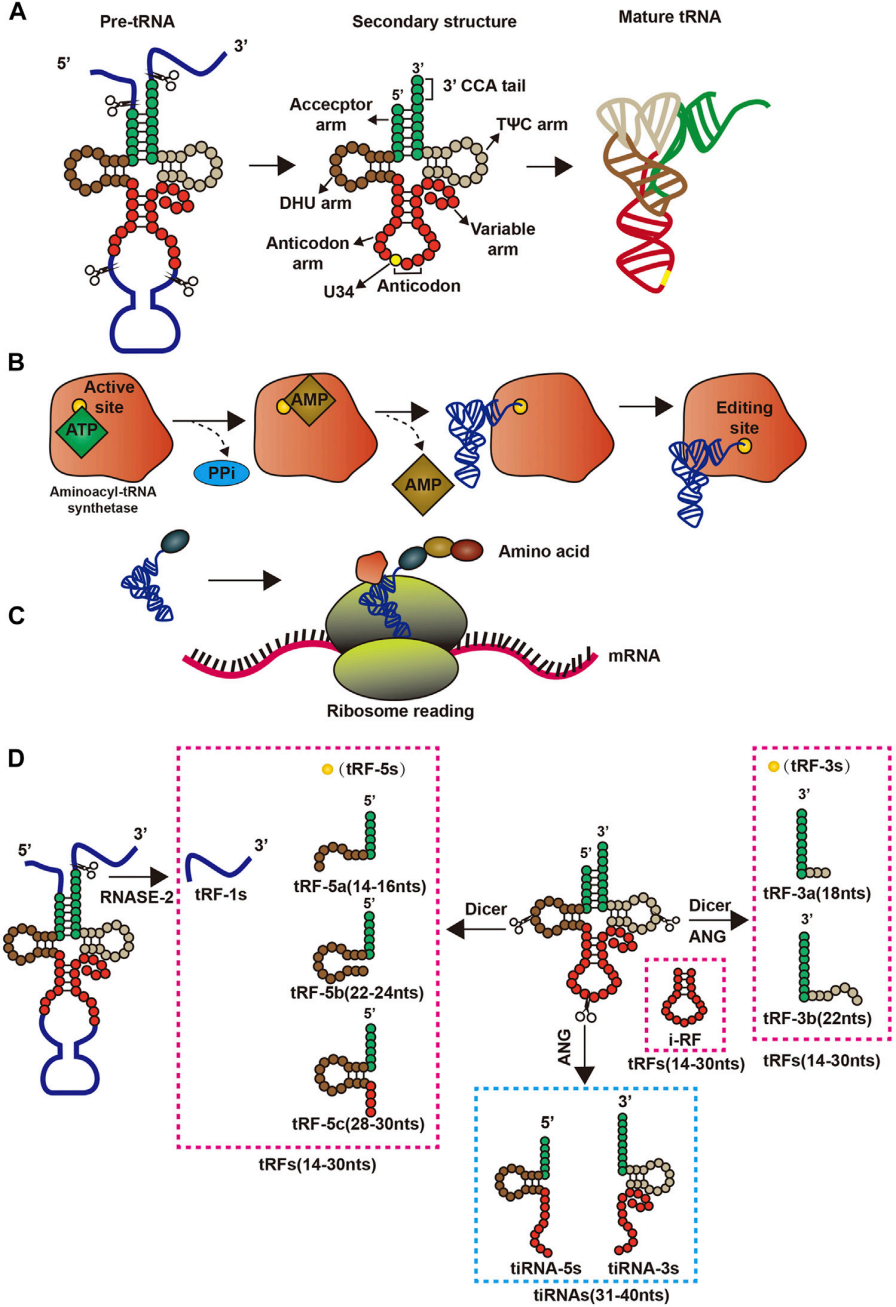
Structure and pre-transcriptional changes in tRNAs. **(A)** Precursor tRNAs forms mature tRNAs; **(B)** Aminoacyl-tRNA synthetase binds amino acids to its corresponding tRNAs by consuming energy; **(C)** tRNAs and ribosomes are involved in the translation process; **(D)** The process by which precursor tRNAs and mature tRNAs form four tsRNAs.

Upon maturation, tRNAs obtains the L-shaped tertiary structure (Kim et al., 1974; Stout et al., 1976; Moras et al., 1980) by means of base build-up and non-Watson-Crick base pairing between the receptor and TΨC arms, and with the D arm and the anti-coding arm (Madison et al., 1966) at the other end (Madison et al., 1966) (Figure 1). This structure allows tRNA to enter the ribosome and convert genetic information into polypeptides. During the translation process, mature tRNAs bind to adenosine covalently at the tail of the unchanging 3′CCA, acting as a connection and can be catalyzed by 20 different aminoacyl-tRNA synthases (Phizicky and Hopper, 2010). Generally, mature tRNAs are highly modified by tRNA-modifying enzymes, which play a vital role in obtaining 3D L-shaped structures and stability, translation start and extension factors, aminoacyl-tRNA synthases and ribosomes, and decoding efficiency and fidelity. Mature tRNAs add amino acids to the two-step reaction through aminoacyl-tRNA synthases, which are activated by ATP to form amino amp, and then add end adenosine to the tRNAs 3′ end (Figure 1). Then, the tRNAs charged with the amino acid interact with the enzyme machinery of the ribosome to decode mRNAs into proteins during translation (Figure 1). Meanwhile, tsRNAs are is a class of non-coding small RNAs produced by mature tRNAs or pre-tRNAs at different sites that are widely present in prokaryotic and eukaryotic transcriptomes, and produced by mature tRNAs or pre-tRNAs at different sites. tsRNAs refers to specific nucleic acid enzymes such as Dicer and angiogenin, especially cells or specific cleavage of tRNAs under certain conditions, such as stress and hypoxia (Levitz et al., 1990; Thompson and Parker, 2009). There are two main types of tsRNAs: tRFs (Keam and Hutvagner, 2015) and the tRNA-derived, stress-induced RNAs (tiRNAs) (Saikia and Hatzoglou, 2015; Shigematsu and Kirino, 2017). tRFs are 14–30 nucleotides (nts) long, and tRFs can be further divided into four categories, namely tRF-5s, tRF-1s, i-tRF, and tRF-3s, depending on their position on tRNAs. tRF-5s come from the 5′ end of mature tRNAs without D-loop and have three subclasses: 1) tRF-5a about 14–16 bases, a cutting site before the D ring; 2) tRF-5b, containing 22–24 bases and cleavage site behind the D ring; and 3) tRF-5c, about 28–30 bases long, has cleavage site before the anticodon ring (Kumar et al., 2014). tRF-3s are rooted in the 3′ end of mature tRNAs and include a CCA parting without the L-loop. They are divided into two subclasses according to size. tRF-3a usually has a cleavage site before the T-ring, while the tRF-3 cleavage site is in the T-ring, and the tRF-3a is usually made up of 18 bases, while the tRF-3 is 22 bases (Kumar et al., 2016). tRF-1s, the third category, is produced at the 3′ end of the pre-tRNAs, and their 5′ ends begin just after the 3′ end of the mature tRNA sequence. Finally i-tRF comes mainly from the middle region of mature tRNAs (Lee et al., 2009).

tiRNAs (31–40 nts in length) are formed by cleaving the tRNA anticodon loop affected by sex hormones and their receptors (Rashad et al., 2020), and have two types: tiRNA-5s and tiRNA-3s (Anderson and Ivanov, 2014; Saikia and Hatzoglou, 2015). Angiogenin, the nuclease responsible for tiRNA formation, generally in the nucleus of cells but under certain conditions can enter the cytoplasm, belonging to the RNase A superfamily (Fu et al., 2009; Li and Hu, 2012). In response to stress, angiogenin is isolated by RNH1 (an angiogenin inhibitor), enters the cytoplasm from the nucleus, and cleaves tRNAs into tiRNA-5s and tiRNA-3s in the cytoplasm (Li and Hu, 2012) (Figure 1).

## Biological Roles and Functions of tRNAs and Their Derivatives

The functions of tsRNAs in different pathways, such as increasing mRNA stability (Saikia and Hatzoglou, 2015; Shigematsu and Kirino, 2017), repressing translation (Kumar et al., 2014; Kumar et al., 2016), regulating ribosome biogenesis (Lee et al., 2009), functioning as novel epigenetic factors (Rashad et al., 2020), promoting RNA reverse transcription (Anderson and Ivanov, 2014), functioning as immune signaling factors (Fu et al., 2009), have recently emerged.

### Regulation of mRNA Stability

**FIGURE 2:**
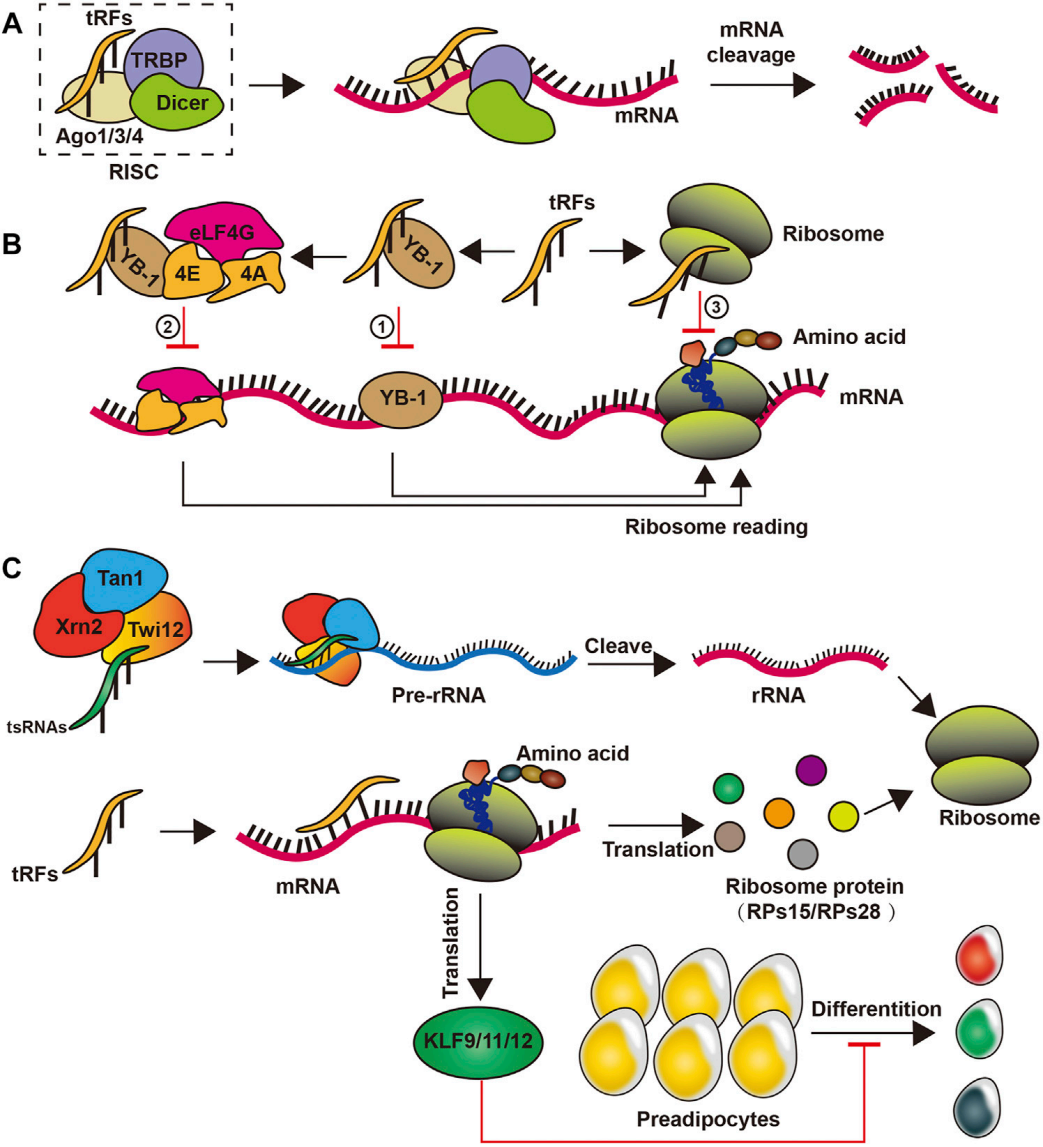
Biogenesis of tsRNAs. **(A)** tsRNAs reduce mRNA stability by binding to a complex such as Ago1/3/4 proteins; **(B)** tsRNAs can inhibit the translation of mRNA by competitive binding of YB-1 or by self-binding of ribosomes; **(C)** tsRNAs promote translation by promoting the formation of rRNAs in ribosomes, while participating in ribosome protein formation to facilitate translation processes and inhibit preadipocytes differentiation.

As miRNAs, tRFs can reduce mRNA stability by mediating target gene deacetylation, thereby promoting mRNA degradation and instability. tRFs prioritizes the inhibition of ribosome proteins and translational initiation or elongation factors of mRNA translation through antisense pairing in *Drosophila melanogaster* (Karaiskos et al., 2015; Babiarz et al., 2008; Eichhorn et al., 2014). In human cells, The GW182 protein inhibits translation and promotes the degradation of target mRNAs, and the tRF-3 target mRNA pairs in the RNA-induced silencing complex associate with GW182 proteins, which means that tRFs can affect the function of RNA-induced silencing complex by regulating the stability of mRNA (Kuscu et al., 2018; Ren et al., 2019). Furthermore, in mature B lymphocytes, tRF-3s derived from tRNA^Gly-GCC^ (referred as CU1276) possess miRNA-like structure and function, thereby repressing mRNA transcripts by destabilizing mRNA, and can inhibit protein translation and the cleavage of a partially complementary target site, thereby suppressing proliferation (Shao et al., 2017a). Although some tRFs have similar functions to miRNAs, the formers have been expressed preferentially bind argonaute1, argonaute3, and argonaute4 to promote RNA-induced silencing complex formation and reduce the stability of mRNA, thus inhibiting mRNA translation, rather than binding argonaute2 like miRNA (Kumar et al., 2014; Haussecker et al., 2010) (Figure 2). In addition, tRF-2s blocks the interplay of Y-box binding protein 1 (YBX-1) and YBX-1 mRNAs by competitively binding to YBX-1. This reduces the stability of these mRNAs, which could subsequently reduce the genetic stability of human breast cancer cell metastasis (Goodarzi et al., 2015) (Figure 2). Moreover, some tRF-3s chimeras have been related to histone mRNAs and can thus affect mRNA stability by competing with stem-ring binding proteins in human cells (Kumar et al., 2014) (Figure 2). Although there is an increased understanding of some of the functions of tRFs in regulating mRNA stability, the functions of tiRNAs remain elusive.

### Downregulation of Translation

Some studies have shown that tiRNAs, mainly tiRNA-5s, could decrease translation speed by 10–15% (Yamasaki et al., 2009). For instance, tiRNA-5s from tRNA^Ala^ and tRNA^Cys^ can form a G-quadruplex-like structure that selectively binds to eIF4G/eIF4A in the translation of the starting complex, thereby inhibiting cap-dependent translation of cellular mRNAs rather than traditionally internal ribosome entry site mediated translation (Ivanov et al., 2011; Ivanov et al., 2014). tiRNAs can also selectively repress the housekeeping components’ translation under stress conditions, thereby reducing cell energy consumption without affecting the generation of pro-survival proteins (Li and Hu, 2012). These studies suggest that tiRNAs are produced to regulate the translation process under stress conditions and are not intended to reduce the level of functional maturity tRNAs affecting mRNA function (Ivanov et al., 2011; Ivanov et al., 2014).

In addition, some researches have proved that tRFs plays a positive role in reducing protein translation behavior. For example, tRF-5s is supposedly involved in new mechanisms underlying the regulation of small RNA in human cell by repressing protein translation through conserved residues in tRNAs present in tRF-5s without the need for complementary target sites in mRNA (Sobala and Hutvagner, 2013). tRF-5s derived from tRNA^Val-GAC^ in *Haloferax volcanii* has been revealed to bind the small ribosomal subunit near the mRNA channel, leading to substitution in the initiation complex and thereby attenuating global translation both *in vivo* and *in vitro* (Gebetsberger et al., 2017).

### Regulation of Ribosome Biogenesis

tsRNAs have recently emerged as important regulators of ribosome biogenesis. Particularly, in the lower organism *Tetrahymena thermophila*, tsRNAs are composition of the precursor ribosomal RNA splicing complex (Couvillion et al., 2012). In *Drosophila*, tsRNAs restrain global translation by impeding ribosome biogenesis. Mechanistically, *Drosophila* argonaute2-bound tsRNAs preferentially inhibit the mRNA translation of ribosome proteins or translational initiation or elongation factors *via* an RNA-like pathway, thus attenuating overall translation (Couvillion et al., 2012; Dou et al., 2019). In addition, tRF-3s can recruit exonuclease Xrn2 and Tan1 protein to form compounds by specifically binding to the Twi12 protein, which cleaves and processes precursor ribosomal RNA to enhance ribosomal RNA synthesis in physiological conditions in *Tetrahymena* (Couvillion et al., 2012). In mammalian cells, tRF-3s from tRNA^Leu-CAG^ bind at least two ribosomal protein mRNAs to itself, such as ribosome proteins 28 and ribosome proteins 15, to promote translation (Kim et al., 2017) (Figure 2). However, the mechanism by which tsRNAs regulate ribosomal biogenesis in human remains to be explored.

### As Novel Epigenetic Factors

Recent studies have unveiled that tsRNAs may function as epigenetic factors to regulate gene expression. Obese rat model under the control of a high-fat diet, increased levels of tRF^Glu-TTC^ directly targeted the transcription factors from the Kruppel-like factor (KLF) family, such as KLF9, KLF11, and KLF12, which are injected themselves into multiplication, apoptosis, differentiation and progress, and significantly suppressed their target mRNA expression, thus preventing the differentiation of preadipocytes (Shen et al., 2019) (Figure 2). Moreover, tRF^Glu-TTC^ suppressed adipogenesis by inhibiting lipids transcription factors’ expression (Shen et al., 2019). tRF-3s of different lengths can block reverse transcription and post-transcription silence by 18 and 22 nts long respectively, thus silence the long terminal repeat reverse transcription transposer (Schorn et al., 2017). Furthermore, Dicer-like 1 processes tRF-5s and then integrates into argonaute1, which, like miRNA, regulates genomic stability by targeting the transcriptional elements in plant *Arabidopsis thaliana* (Martinez et al., 2017). Intriguingly, tsRNAs from high-fat diet male sperm injected into normal fertilized eggs of mature mouse sperm caused gene expression changes in the early embryo and triggered islet metabolic pathways independent of DNA methylation in CpG-enriched regions. This proves that tsRNAs may be a paternal epigenetic factor in the intergenerational inheritance of metabolic diseases affected by a mediated diet (Chen et al., 2016).

### Upregulation of RNA Reverse Transcription

tsRNAs can also act as an agonist for viral reverse transcription and promote the viral reverse transcription process in various ways. For example, tRF-3s can combine with the primer-binding site of human T cell leukemia virus type 1 RNA to initiate reverse transcription and promote viral synthesis of HIV-infected host cells (Ruggero et al., 2014). Meanwhile, the respiratory syncytial virus infection induces the angiotensincutting2 tRNAs to produce tiRNAs, thus triggering stress response in host cells. respiratory syncytial virus uses host tiRNAs as primers to promote its replication and improve the infection efficiency (Wang et al., 2013; Deng et al., 2015; Zhou et al., 2017).

Moreover, host cellular proteins can regulate retroviral replication by binding to tRNAs, thereby affecting all steps in the viral life cycle. In certain circumstances, aminoacyl-tRNA synthetases bind tRNAs and link them with their corresponding amino acids and cognate aminoacyl-tRNA synthetases to facilitate tRNA primer selection, thus promoting viral reverse transcription (Jin and Musier-Forsyth, 2019).

### Immune Regulation

**FIGURE 3:**
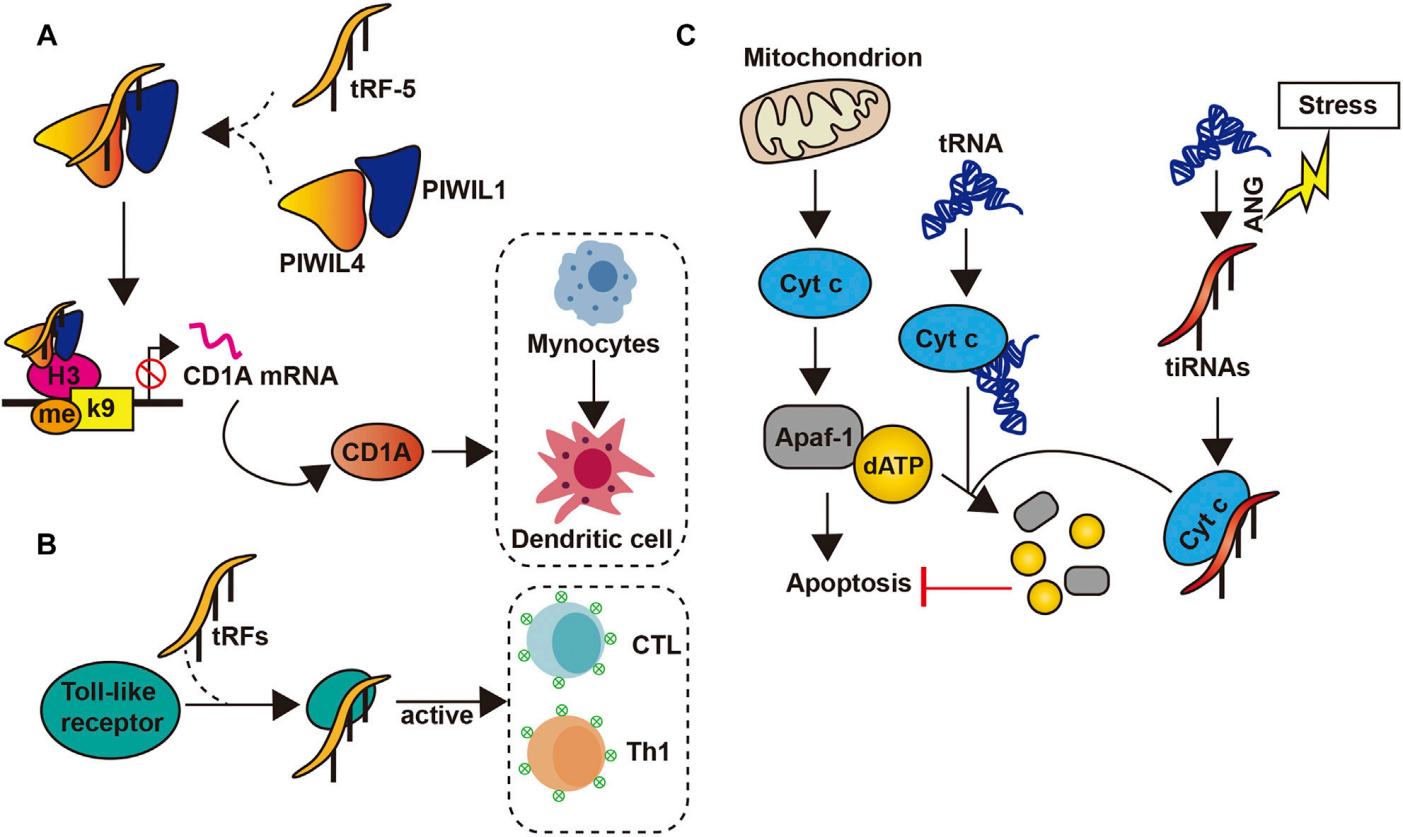
tsRNAs’ functions. **(A)** tsRNAs inhibit the transcription of the CD1A gene by forming a complex with PIWIL1/4, thereby inhibiting the conversion of Mynocytes to Thedritic cell; **(B)** tsRNAs can be combined with toll-like receptor to activate CTL and Th1; **(C)** The interaction between tsRNAs and Cty c inhibits the apoptosis process.

Studies have also revealed the potential role of tsRNAs as novel immune factors. tsRNAs are highly and stably expressed in hematopoietic and lymphatic organs and blood compared with other tissues (Dhahbi, 2015). This suggests that tsRNAs may participate in the immune process. In addition, when the body is in an acute inflammatory state, tsRNAs levels in the blood increase rapidly, particularly in the sera of mice and monkeys with acute and chronic hepatitis B and active hepatitis B virus infection, as well as chimpanzees with chronic viral hepatitis (Zhang et al., 2014; Selitsky et al., 2015). Likewise, tRF-5s derived from tRNA^Glu^ can lead to the inhibition of CD1A expression by compounding with PIWIL4 and PIWIL1, which was able to promote the maturation of monocytes into dendritic cells (Zhang et al., 2016) (Figure 3). Moreover, tsRNAs can also activate the immune response of Th1 and cytotoxic T lymphocyte by interacting directly with toll-like receptors (Wang et al., 2006) (Figure 3).

In addition, the specific nucleoside motifs of tRNAs may be a structural determinant of innate immune recognition. For example, the interaction between the human tRNA^Ala^ stem loop and the D and T rings of tRNA^His^ may be epitopes of autoantibodies in the sera of patients with idiopathic inflammatory myopathy (Bunn and Mathews, 1987), and the anti-adenovirus infection-induced tRNAs fungal protective cell therapy (Alvarado-Vásquez et al., 2005). Finally, the major histocompatibility complex contains the largest tRNA gene cluster in human, which also coexist with immune-related functions are co-located. This may imply the role of tRNA in the immune system (Horton et al., 2004). These findings overall support the proposition that tRNAs may act as immune signaling molecules.

### Other Mechanisms of Action

Both mitochondrial and cytosolic tRNAs have been shown to bind to cytochrome c. This binding inhibits the interaction between cytochrome c and apoptotic protease activating factor-1, thus blocking activating factor-1 oligomerization and caspase activation, which eventually preventing apoptosis (Mei et al., 2010). Another study revealed that under high osmotic pressure or stress, angiogenin-induced tiRNAs can inhibit apoptotic formation and activity by binding cytochrome c to form a ribonucleoprotein complex (Saikia et al., 2014) (Figure 3). Moreover, tsRNAs can also regulate micro-organisms found in human. In the oral cavity, the presence of *Fusobacterium nucleatum* triggers the release of tsRNAs, which may inhibit the growth of the former by interfering with the biosynthesis of bacterial proteins (He et al., 2018).

At present, studies exploring the function of tRNAs are only emerging. Future researches are expected to reveal the further regulatory role of tRNAs and tsRNAs in biological functions.

## tRNAs and tsRNAs in Human Cancer

tRNAs dysregulation have been detected in a variety of cancers, including breast cancer (Fang et al., 2017), lung cancer (Lu et al., 2009), melanoma (Phizicky and Hopper, 2010), prostate cancer (Olvedy et al., 2016a), and tRNA has been revealed to be regulated by oncogenes and tumor-suppressor genes. Particularly, oncogenes *Ras* (Wang et al., 1997) and *c-myc* (Gomez-Roman et al., 2003) can promote the expression of RNA polymerase III, whereas suppressor genes Rb (White et al., 1996) and p53 (Crighton et al., 2003) can inhibit its transcription. This leads to a serious imbalance of tRNA expression in cancers. Abnormally expressed tRNA can also promote cell proliferation and inhibit cell apoptosis, thus promoting tumor progression (Pavon-Eternod et al., 2013; Kwon et al., 2018).

In addition, the tRNA modification system can initiate tumorigenesis by directly affecting cellular processes associated with characteristic cancer cell phenotypes, such as increased proliferation, metastasis potential, and stem cell survival (Endres et al., 2019). Further, the tRNA modification system is also a key component of carcinogenic signaling pathways (Endres et al., 2019). In various cancers, the tRNA modifying enzyme increases the modification of a specific tRNA, thereby altering the preference of the tRNA codon. This results in increased levels of proteins corresponding to these mRNAs was found to be rich in a particular subset of the new “preferred” codons (Novoa et al., 2012; Novoa and Ribas De Pouplana, 2012).

Mutations in mitochondrial tRNA (mt-tRNA) have been confirmed that increased tumorigenic relates to the invasive phenotypes (Amuthan et al., 2001). These mutations can seriously affect the tertiary structure of mt-tRNAs, thus severely damaging the synthesis of mitochondrial proteins (Grzybowska-Szatkowska and Slaska, 2012). At present, the involvement of mt-tRNA mutations in the carcinogenesis of breast and lung cancers has been confirmed (Lu et al., 2009; Meng et al., 2016).

Several studies have also reported that tsRNAs, a derivative of tRNA, is dysregulated in a variety of cancers and may play a carcinogenic or anti-cancer role (Pekarsky et al., 2016; Balatti et al., 2017). In breast cancer, many studies have shown that cancer genes can regulate the expression of tsRNAs, which may also be a key effector molecule in cancer gene regulation (Balatti et al., 2017).

**FIGURE 4:**
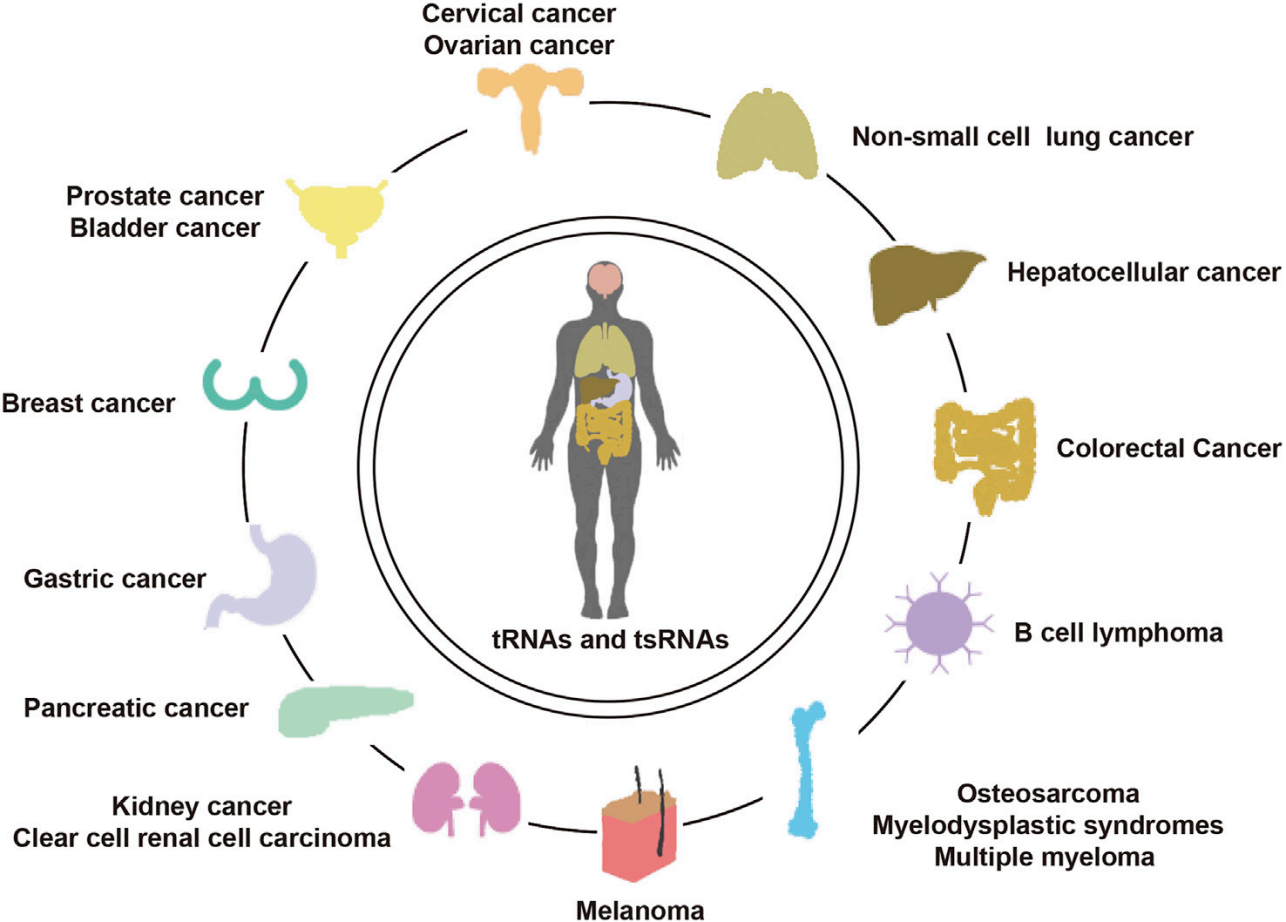
Landscape of tRNAs in human diseases.

While current knowledge of tRNAs and tsRNAs in cancer is still in its infancy, their potential applications in the improvement of fresh biomarkers and original healthful strategies for the diagnosis, monitoring, predicting, and treating cancer cannot be understated. We have summarized the recent literature on the functions and mechanisms of cancer-associated tRNAs in Table 1 (Figure 4).

**TABLE 1:** Summary of human cancer-associated tRNAs and tsRNA.

tRNAs and tsRNA	Function	Role	Cancer type	References
tRF^Glu-YTC^	Destabilization of YB-1 bound oncogenic transcripts then suppresses cell proliferation and cancer metastasis	Tumor suppressor	Breast cancer	Goodarzi et al. (2015)
tRF^Asp-GTC^				
tRF^Gly-TCC^				
tRF-1001	Promotes cell proliferation	Tumor promoter	Prostate cancer	Lee et al. (2009)
tiRNA^Asp-GUC^	Sex hormone-dependent production, promote cell proliferation	Tumor promoter	Breast cancer and prostate cancers	Honda et al. (2015)
tiRNA^His-GUG^				
tiRNA^Lys-CUU^				
tiRNA^Leu-CAG^-5	Promotes cell proliferation and G_0_/G_1_ cell cycle progression, greatly upregulates in stage III and stages IV cases and relates with the development of stage	Tumor promoter	Non-small cell lung cancer	Shao et al. (2017a)
tRF/miR-1280	Inhibits cell proliferation and tumor growth through inhibiting Notch signaling pathway by targeting JAG2	Tumor suppressor	Colorectal Cancer	Huang et al. (2017)
CU1276/tRF-3018	Associates with Argonaute proteins and represses endogenous RPA1, suppresses proliferation and modulates the molecular response to DNA damage	Tumor suppressor	B cell lymphoma	Maute et al. (2013)
tRF^Ser-GCT^	Unknown	Unknown	Breast cancer	Telonis et al. (2015)
tRF^Ser^	Cleavage of tRNAs during stress	Unknown	Hepatocellular cancer	Fu et al. (2009)
tRF^Lys3^	Combine with AGO2 and target HIV primers with binding sites	Unknown	Cervical cancer	Yeung et al. (2009)
tRF^His-GTG^	Associates with AGO2 and down-regulate target genes by transcript cleavage	Unknown	B cell lymphoma	Li et al. (2012)
tRF^Leu-CAG^				
tiRNA^Ala^	Inhibits protein synthesis and triggers the phospho-eIF2α independent assembly of stress granules	Promoting tumor	Osteosarcoma	Ivanov et al. (2011)
tiRNA^Cys^				
tRF^Val^	Induces the assembly of cytoprotective stress granules	Unknown	Osteosarcoma	Emara et al. (2010)
tRF^Gln^	Inhibits the process of protein translation without the need for complementary target sites in the mRNA	Tumor suppressor	Cervical cancer	Sobala and Hutvagner, (2013)
cand14	Primarily associates with AGO3 and AGO4, RNA silencing by targeting luciferase reporter gene	Unknown	Kidney and colorectal cancer	Haussecker et al. (2010)
cand45				
tiRNA^Val^	Associates with response to the treatment of DNA methyltransferase inhibitors	Unknown	MDS	Guo et al. (2015)
Multiple tRFs	Overexpresses in metastatic tissues, potential diagnostic and prognostic biomarkers	Unknown	Prostate cancer	Martens-Uzunova et al. (2012)
tRF-544	High expression ratio of tRF-315/tRF-544 predict poor PFS	Unknown	Prostate cancer	Olvedy et al. (2016b)
tRF-315				
tRF^Val-AAC^	Greater downregulation in advances and less differentiates in ccRCC tissues	Tumor suppressor	Clear cell renal cell carcinoma	Nientiedt et al. (2016), Zhao et al. (2018)
tiRNA^Leu-CAG^-5				
tiRNA ^Arg-CCT^-5				
tiRNA ^Glu-CTC^-5				
tiRNA ^Lys-TTT^-5				
ts-46	ts-47: upregulated with KRAS mutation	Tumor suppressor	Lung cancer, breast cancer	Balatti et al. (2017)
ts-47	ts-46: upregulated with PIK3CA mutation in breast cancer cells; inhibition effect on colony formation in H1299 and A549 cell lines			
ts-53	ts-53: reduce lung cancer colony formation through exogenous expression; ts-53, ts-101: act as miRNAs and piRNAs by their interaction with argonaute and Piwi proteins	Tumor suppressor	Lung cancer, CLL	Pekarsky et al. (2016)
ts-101				
miRNA-1236-3p	Suppresses the proliferation, migration, and invasion capacity of cancer cells	Tumor suppressor	Hepatocellular cancer, ovarian cancer, bladder cancer, gastric cancer	An et al. (2019)
tRF-03357	Promotes cell proliferation, migration, and invasion	Tumor promoter	High-grade serous ovarian cancer	Zhang et al. (2019b)
tRNA^Leu^	Promotes cell proliferation and transformation	Tumor promoter	ER + breast	Fang et al. (2017)
			Cancer	
tRNA^Leu^	Initiated tumorigenesis	Tumor promoter	Triple-negative breast cancer	Khattar et al. (2016)
tRNA^Tyr^				
tRNA^Leu-CAG^	Increases protein synthesis and proliferative ability of cancer	Tumor promoter	Her2(ErbB2)-positive breast	Kwon et al. (2018)
mt-tRNA^Asp^	Involved in the carcinogenesis of breast cancer	Tumor promoter	Breast cancer	Meng et al. (2016)
tRNA^Arg-CCG^	Promotes metastasis and invasion	Tumor promoter	Breast cancer	Goodarzi et al. (2016)
tRNA^Glu-UUC^				
methionine tRNA	Advances cancer cell migration, invasiveness, and lung colonization capacity	Tumor promoter	Melanoma	Birch et al. (2016)
tRNA^Arg^	TERT promotes cancer cell	Tumor promoter	Melanoma	Khattar et al. (2016)
tRNA^Ala^	Proliferation by augmenting tRNA expression			
tRNA^Asn^				
tRNA^Cys^				
tRNA^Lys^				
tRNA^Glu^				
tRNA^Thr^				
tRNA^Arg^	The oncoproteins E6 and E7 stimulates tRNA transcription	Tumor promoter	Cervical cancer	Daly et al. (2005)
tRNA^Sec^				
High levels of tRNA abundance	Increases translation of highly active proteins	Tumor promoter	Multiple myeloma	Zhou et al. (2009)

### Breast Cancer

**FIGURE 5:**
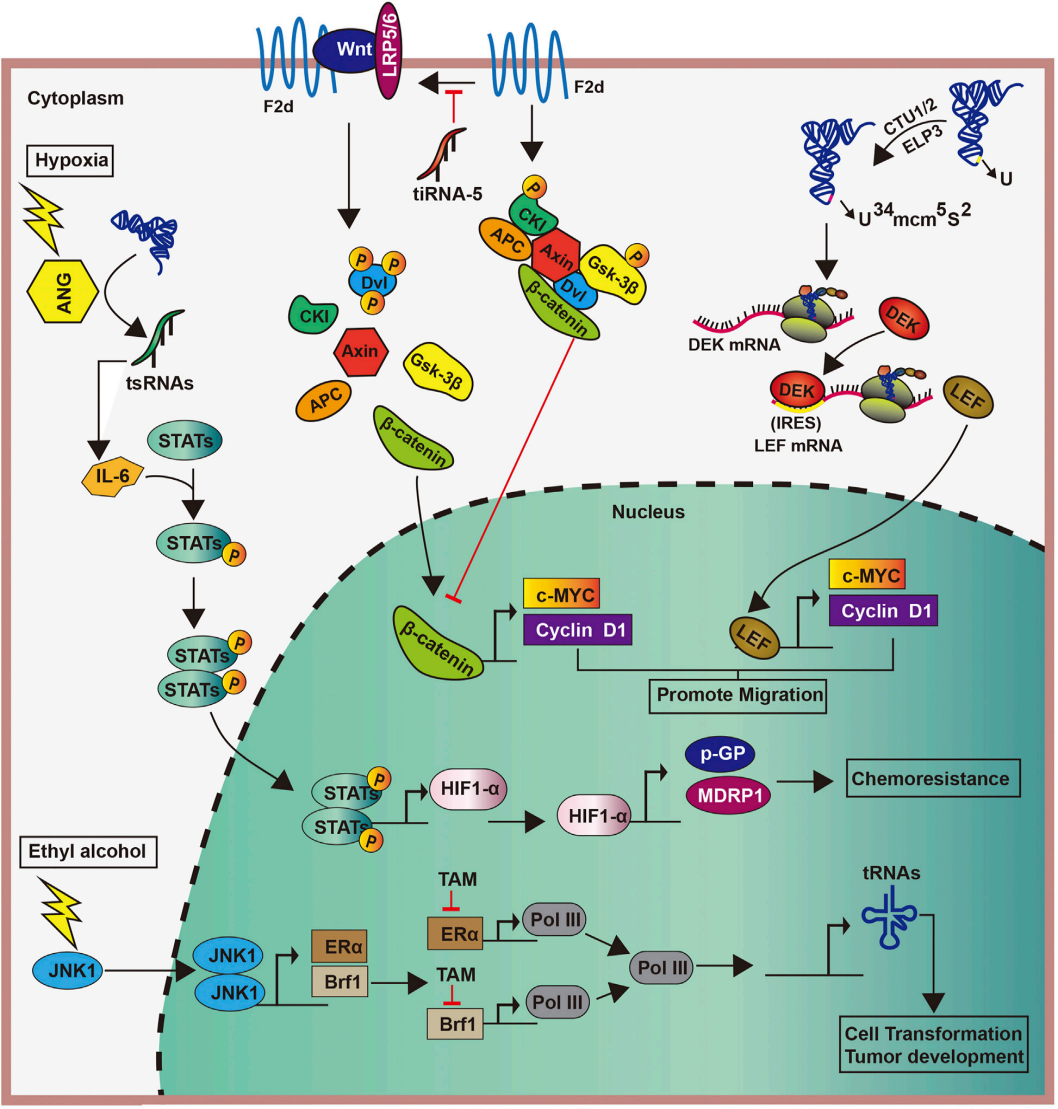
Roles of tRNAs and tsRNAs in breast cancer.

Different factors can induce the abnormal expression of tRNAs in breast cancer, thereby promoting tumor progression. For example, the stimulation of ethanol activates c-Jun N-terminal kinase 1, which promotes the proliferation of Brf1 and ERα. Subsequently, the interaction between Brf1 and ERα upregulates *Pol III* gene transcription to enhance the production of tRNA, ultimately leading to the development of breast cancer. However, tamoxifen can hold in the incident of breast cancer by containing the effects of Brf1 and ERα, also indirectly inhibits the generation of tRNA (Fang et al., 2017) (Figure 5). Similarly, the TATA box-binding protein human Maf1 and the oncogene *Ras* can promote the transcription of tRNAs by targeting RNA pol Ⅲ, particularly the Brf1 subunit of TFⅢ B factor, thereby promoting tumor progression (Wang et al., 1997; Shen et al., 1998; Rollins et al., 2007; Johnson et al., 2008).

Enzymes catalyzing tRNA modifications play significant roles in the biological processes in breast cancer (Towns and Begley, 2012; Frye and Watt, 2006; Delaunay et al., 2016). In human, the overexpression of the U34-modifying enzymes Elp3 and Ctu1/2 directly promoted the translation of the oncoprotein DEK by catalyzing the mcm^5^s^2^-U34 tRNA modification. Increased DEK can then bind the LEF1 internal ribosome entry site sequence, thereby increasing the translation of the oncogenic LEF-1 mRNA and promoting the invasion and metastasis of breast cancer cells (Delaunay et al., 2016) (Figure 5). Furthermore, the high expression of tRNA^Arg-CCG^ and tRNA^GIu-UUC^ in breast cancer can promote the invasion and metastasis of cancer cells by directly upregulating the expression of EXOSC2 and enhancing that of GRIPAP1 (Goodarzi et al., 2016).

In a study cohort of Polish women with breast cancer, the tertiary structure of mt-tRNAs was affected by genetic mutation, resulting in the severe impairment of the mitochondrial protein synthesis, and thus affecting cell proliferation (Grzybowska-Szatkowska and Slaska, 2012). Further, mutations in mt-tRNAs are participated in the carcinogenesis of breast cancer, such as mt-tRNA^Asp^ (Meng et al., 2016), but the specific mechanism of mtRNA mutation in cancer is unknown.

Similarly, tiRNAs are abnormally expressed in breast cancer and are involved in tumorigenesis (Goodarzi et al., 2015; Honda et al., 2015; Balatti et al., 2017). Decreased abundances of 26 specific circulatory tiRNAs from the tRNA^Gly^, tRNA^Glu^, and tRNA^Lys^ heterogenous receptors was observed in ER-positive breast cancer (Dhahbi et al., 2014). The same study have suggested that inflammatory breast cancer is associated with an increase in tiRNA^Ala^ (Dhahbi et al., 2014). Some tiRNAs, such as tiRNA^Asp^-5 and tiRNA^His^-5, are significantly overexpressed in breast cancer, and the knockout of tiRNA-5s can inhibit tumor proliferation (Honda et al., 2015). These findings provide sufficient evidence of the involvement of abnormal tiRNAs expression in the course of breast cancer.

The connection of tsRNAs in breast cancer progression has also been confirmed by several studies. First, the anomalous demonstration of tiRNAs has been sighted at some stages of the carcinogenesis process [19]. The downregulation of tiRNA^Val^-5 in the serum is positively associated with lymph node metastasis and cancer stage progression, whereas its overexpression inhibits malignant cell activity (Mo et al., 2019). Meanwhile, the expression of tRF-3s are strongly downregulated in invasive advanced breast cancer, whereas that of tRF-1s were raised in an advanced cancer cell, thus suggesting that tRF-3s and tRF-1s may be related to advanced pathological changes in cancer (Balatti et al., 2017). Second, tsRNAs can regulate breast cancer progression by affecting gene transcription. For example, tRFs from tRNA^Glu^, tRNA^Asp^, tRNA^Gly^, and tRNA^Tyr^ contend with YB-1 for the transcription of endogenous cancer genes, thereby undermining the stability of transcriptions of proto-oncogene and reducing their expression. This subsequently inhibits breast cancer progression (Goodarzi et al., 2015). Similarly, in breast cancer cells, tRFs derived from tRNA^Tyr^, tRNA^Asp^, tRNA^Gly^, and tRNA^Glu^ can inhibit tumor progression by displacing the 3′-UTRs of multiple oncogenic transcripts from the RNA-binding protein YBX-1, thus reducing their stability (Goodarzi et al., 2015). Another study showed that tiRNA^Val^-5 leads to the inhibition of *c-myc* and *cyclinD1* by downregulating the FZD3-Wnt/β-catenin axis, which inhibits the progression of breast cancer (Mo et al., 2019) (Figure 5). Third, the impairment of the tiRNAs production also affect cancer progression. estrogen and its receptors promote the angiogenin cutting mature tRNA anticodon ring, thus producing large amounts of tiRNAs in ER-positive breast cancer. This accumulation makes for cells proliferating, which may promote tumor occurrence and tumor growth (Honda et al., 2015). Meanwhile, in a hypoxic environment, angiogenin induces the production of tsRNAs, and tsRNAs then interact with interleukin-6 to promote the phosphorylation of signal transducers and activators of transcription proteins. This promotes the transcription of the hypoxia inducible factor-1α, as well as those of multidrug-resistant genes and glycolytic proteins, ultimately leading to cytochemical resistance (Cui et al., 2019) (Figure 5). These studies suggest the varied functions of tsRNAs in cancer pathogenesis.

### Lung Cancer

**FIGURE 6:**
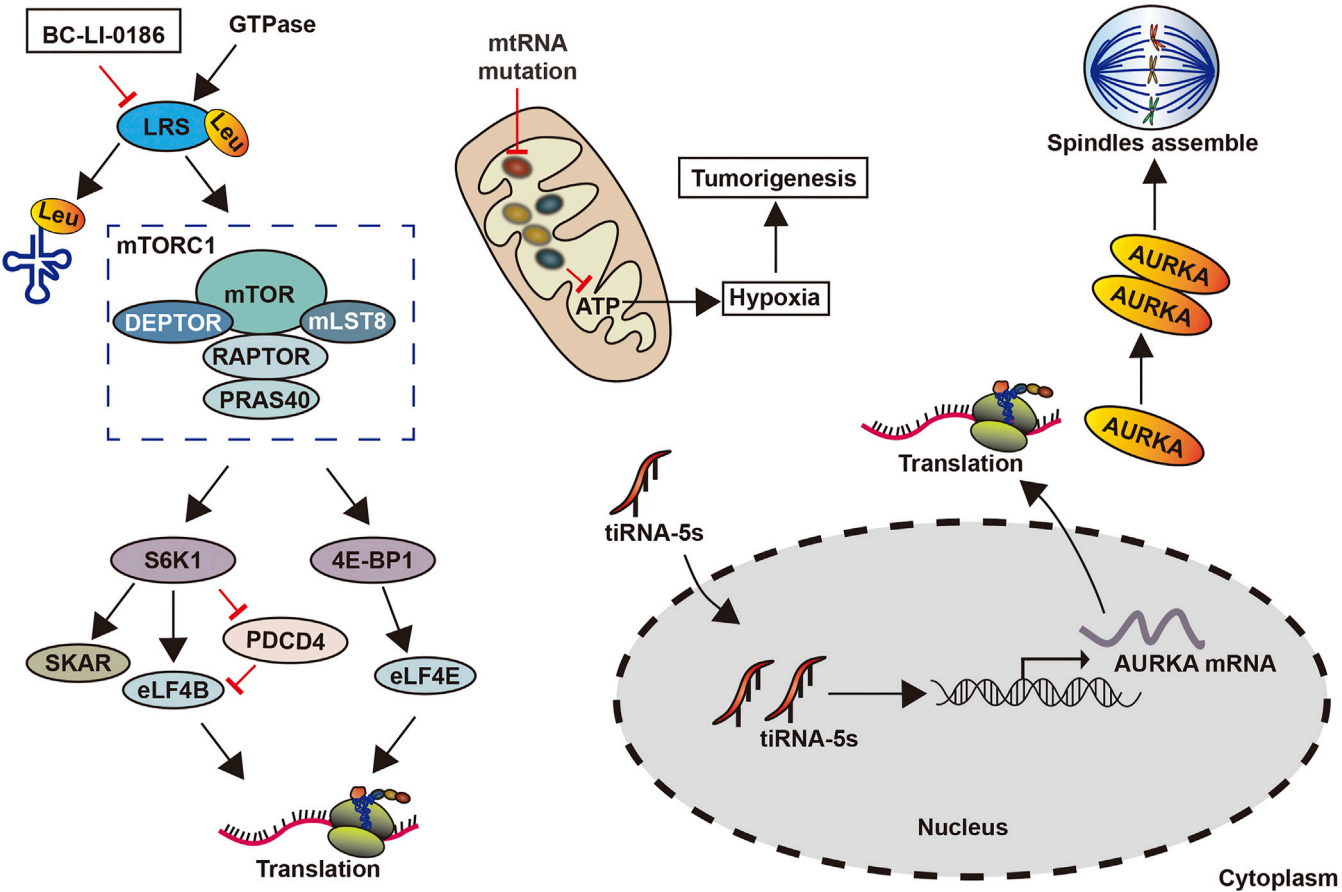
Roles of tRNAs and tsRNAs in lung cancer.

mt-tRNA gene mutations have been found to promote the development of lung cancer (Lu et al., 2009). These mutations damage the secondary structure of tRNAs, thereby affecting post-transcriptional modifications and aminoacylation, which can change the specificity, stability, or affinity of tRNAs (Brulé et al., 1998). Further, these mutations caused a decrease in mitochondrial protein synthesis and the cellular inability to reach the respiratory phenotypes and ATP thresholds required by normal cells to promote lung cancer (Lu et al., 2009) (Figure 6). The drug BC-Li-0186 when combined with leucyl-tRNA synthetase inhibited its activity, reduced the abundance of tRNA-carrying leucine, and prevented leucyl-tRNA synthetase mediated the non-classical mammalian target of rapamycin complex 1. This ultimately restrains the development of non-small cell lung cancer (Kim et al., 2019) (Figure 6).

tsRNAs have also been associated with lung cancer development through its regulation of the biological behavior of cells. For instance, miR-4521, acts as an inhibitor in CLL, has been revealed to be a tsRNA (ts-4521), which is lowered and mutated genes in lung cancer. Meanwhile, a reduction in ts-4521 has been shown to support tumor movement by the cell proliferation-related pathways’ activation and inhibiting apoptosis-related pathways in cancer cells (Phizicky and Hopper, 2010; Pekarsky et al., 2016). In addition, tsRNAs can regulate the demonstration of oncogenes. tRF‐Leu‐CAG is highly expressed in non-small cell lung cancer tissues, promoting tumor cell proliferation and cell cycle progression by upregulating the oncogene *AURKA* (Shao et al., 2017b) (Figure 6). However, how to mediate other signaling pathways through *AURKA* remains unclear.

### Melanoma

**FIGURE 7:**
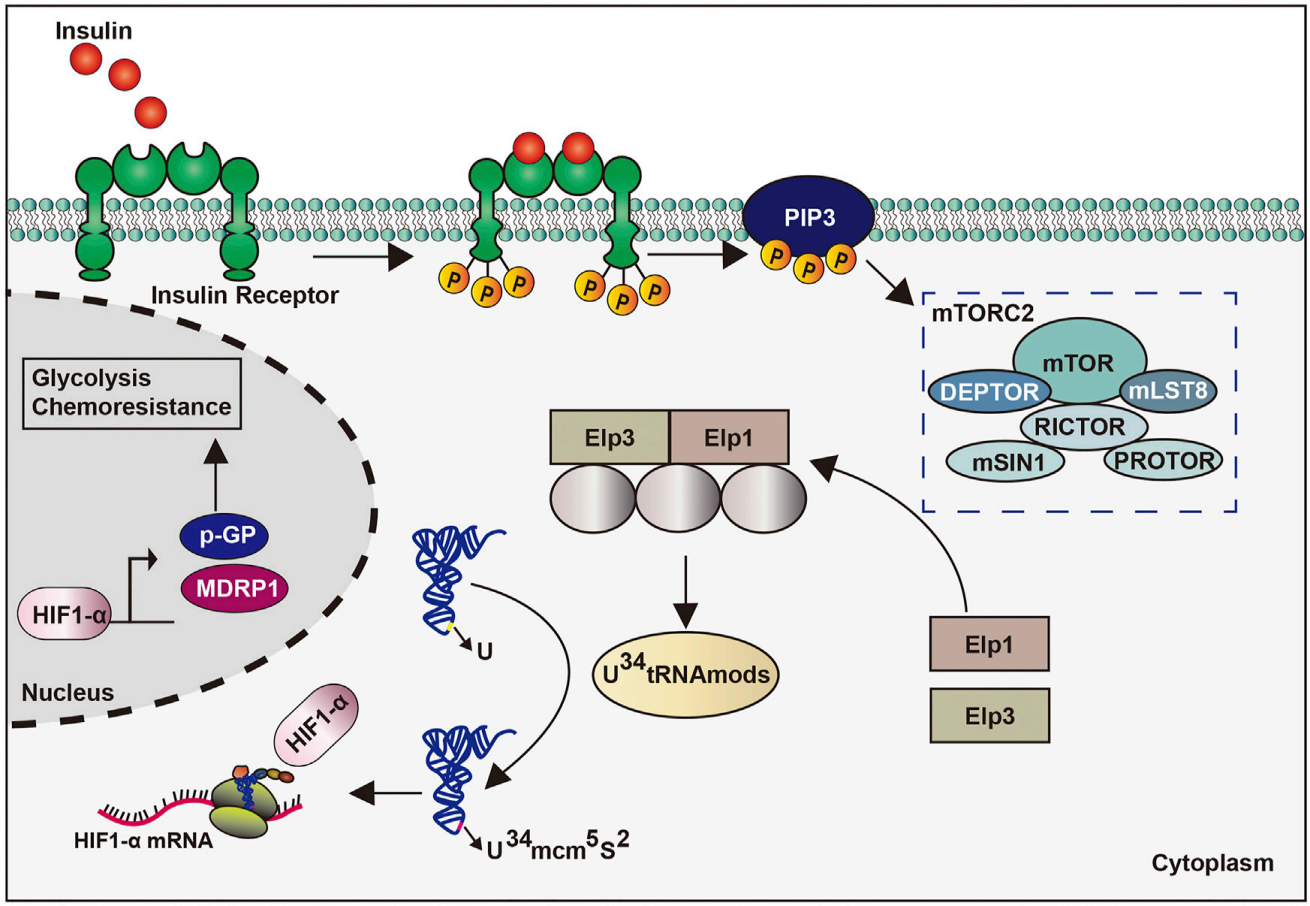
Roles of tRNAs and tsRNAs in melanoma.

Melanoma is highly malignant and accounts for the majority of skin tumor deaths. The roles of tRNAs and tsRNAs in its melanoma have also been investigated. The overexpression of the promoter methionine tRNA gene promoted tumor growth and angiogenesis in mouse melanoma cells, as well as an increase in cancer cell migration, invasion, and lung colonization, thereby resulting in increased metastasis potential (Phizicky and Hopper, 2010; Clarke et al., 2016). Its upregulation in cancer-associated fibroblasts accelerated the secretion of stromal cells, especially type-II collagen, thus facilitating tumor growth and metastasis (Phizicky and Hopper, 2010) (Figure 7).

In a recent study, the accurate translation of hypoxia inducible factor 1 *α* mRNA in melanoma requires the participation of 34 uridine tRNA-modifying enzymes to adapt to a metabolic environment that is not conducive to growth conditions. The particular translation reprogramming of which relies partly on mTORC2-mediated enzymatic phosphorylation to modify the anti-codon of tRNA. Further, enhanced codon dependence on hypoxia inducible factor 1 *α* translation can promote glycolytic metabolism and the proliferation of melanoma cells (Mcmahon and Ruggero, 2018) (Figure 7). However, the specific regulatory mechanism of tRNAs in melanoma needs further validation, whereas the role of tsRNAs in melanoma has yet to be studied.

### Other Cancers

**FIGURE 8:**
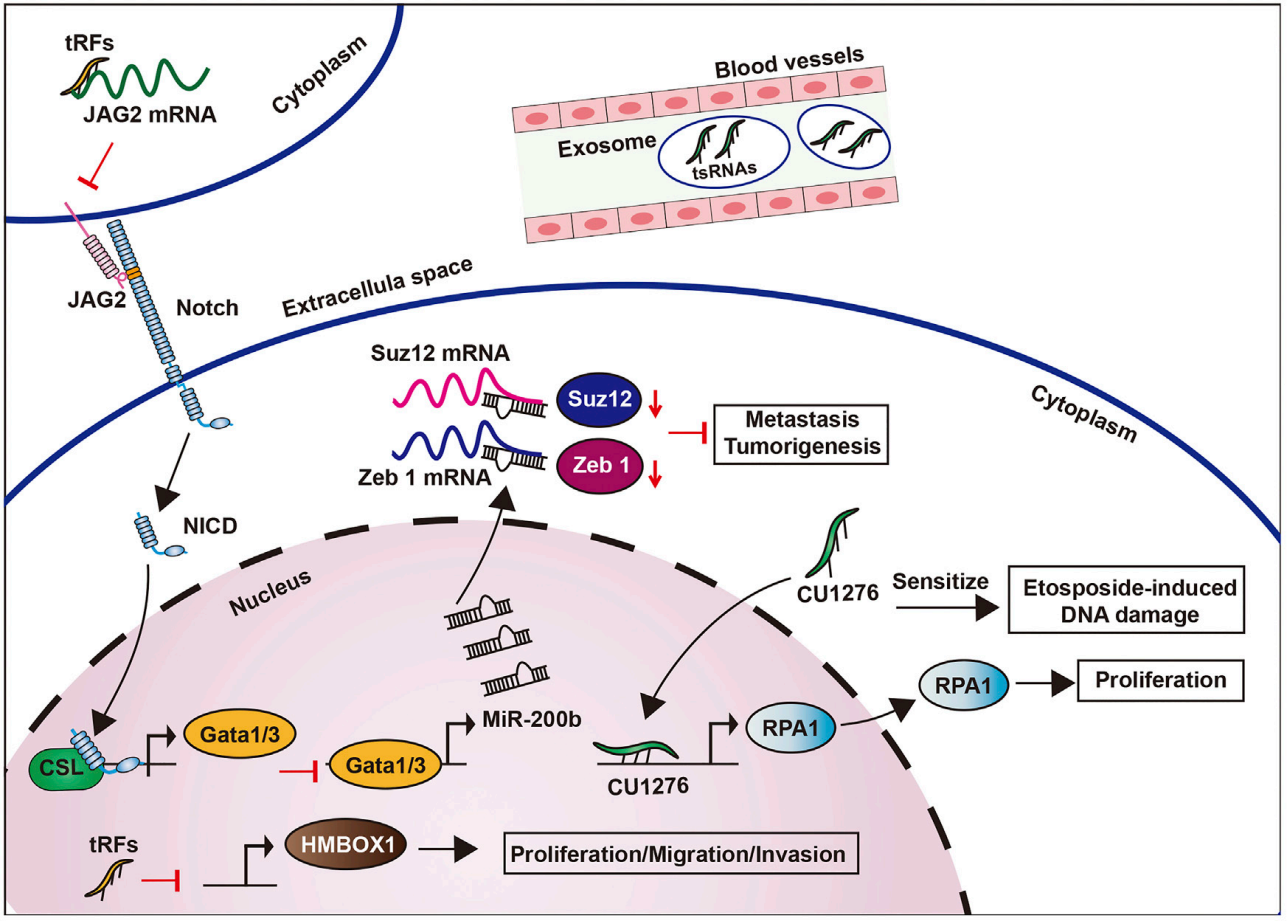
Roles of tRNAs and tsRNAs in other cancers.

tRNAs and tsRNAs have also been implicated in the biological processes of liver (Selitsky et al., 2015) and prostate cancers (Olvedy et al., 2016b). In liver cancer, tsRNAs tRNA^Val-TAC-3^, tRNA^Gly-TCC-5^, tRNA^Val-AAC-5^, and tRNA^Glu-CTC-5^ were significantly increased in plasma exosomes (Selitsky et al., 2015) (Figure 8). Meanwhile the expression of tiRNA^Arg-CCT^-5, tiRNA^Glu-CTC^-5, tiRNA^Leu-CAG^-5, and tiRNA^Lys-TTT^-5 was downregulated in clear-cell renal-cell carcinoma, suggesting a potential role as a tumor suppressor (Zhao et al., 2018). Moreover, the relative abundance of tiRNA^Gly^ is 50–60% lower in hepatitis B virus and Hepatitis C virus-related cancers than normal liver tissue (Selitsky et al., 2015). These findings suggest that tRNAs and tsRNAs may have opposite effects in liver cancer and clear-cell renal-cell carcinoma and may thus be used as new diagnostic biomarkers.

tsRNA (CU1276) modulated DNA damage response and suppressed cell proliferation *via* the inhibition of RPA1, which is an endogenous single-stranded DNA binding protein, in B cell lymphoma cells (Maute et al., 2013) (Figure 8). Meanwhile, tRNA^Leu^ and pre-miRNA derived from tRF/miR-1280, can suppress the growth and transfer of colorectal cancer by inhibiting Notch signaling pathways. Particularly, tRF/miR-1280 can target the Notch ligand jagged 2 (JAG2) to repress Notch signaling pathways, which in turn inhibits the cancer stem cell phenotypes by inhibiting direct transcription of Gata1/3 and miR200b genes, thus inhibiting tumorigenesis and metastasis (Huang et al., 2017) (Figure 8).

In high-grade serous ovarian cancer, upregulated tRFs can promote protein phosphorylation, transcription, cell migration, cancer pathways, MAPK, and Wnt signaling pathways, as well as regulating HMBOX1 to attack human ovarian cancer cells (Zhang et al., 2019a). This proves that tRFs play a role as a regulatory factor for cancer development in serous ovarian cancer (Figure 8). In prostate cancer, 589 differentially expressed tRFs have been detected, suggesting its potential as a biomarker (Olvedy et al., 2016b). Similarly, tRF-1001 from tRNA^Ser^ was eminently expressed in prostate cancer, whereas its knockdown suppressed DNA biosynthesis and cell proliferation (Anderson and Ivanov, 2014).

## Clinical Application of tRNAs and tsRNAs in Cancer

Although tsRNAs and tRNAs function in the human body are still in the exploratory stage, the potential it has shown as a cancer marker is not to be underestimated. Next, we will discuss its role in clinical diagnosis and treatment.

### As a Diagnostic Biological Marker

Cancer is the most dangerous disease known. In many cases, patients with cancer are often unaware of their early stages, and when clinical symptoms appear, the cancer is in its late stages. Finding reliable and sensitive diagnostic molecular markers is researchers have been struggling to find. Recently, the function of tRNAs and tsRNAs in the human body has gradually received clinical attention and shown great molecular marker potential.

Previous studies have shown that tsRNAs can be detected in serum and urine stabilized (Romaine et al., 2015; Santos et al., 2019). Now, with the continuous development of high-throughput sequencing technology, different types of tsRNAs are constantly detected and isolated in body fluids, and their functions are gradually reflected. For example, there are differences in abundance between prostate cancer and renal transparent cell carcinoma and tsRNAs in normal prostate tissue (Hayes et al., 2014; Zhao et al., 2018). Subsequently, many studies have found tRNAs and tsRNAs disorders may be related to the regulation of tumor genes and tumor suppressor genes, while the abnormal expression of tRNAs and tsRNAs can promote cell proliferation and inhibit cell apoptosis, further promoting cancer progression (Pavon-Eternod et al., 2013; Kwon et al., 2018). In breast cancer, ethanol stimulation can promote the proliferation of Brf1 and ER+, thereby enhancing their interaction, and enhance the production of tRNA by Pol III gene transcription, further promoting tumor progression (Fang et al., 2017). Meanwhile many studies have shown that cancer genes can regulate the expression of tsRNAs and tsRNAs may also be a key effector molecule of cancer gene regulation (Zhu et al., 2019). On the other hand, in hepatocellular carcinoma, tsRNAs tRNA^Val-TAC-3^, tRNA^Gly-TCC-5^, tRNA^Val-AAC-5^, and tRNA^Glu-CTC-5^ are significantly elevated in plasma exosomes, which may play a transcriptional role (Selitsky et al., 2015). These characteristics are sufficient to demonstrate the great potential of tsRNAs to become a biomarker of tumors. But there is still less research on tsRNAs, and hopefully more people will look to it in the future so that tsRNAs can really play its clinical role and contribute to the cause of human medicine.

### The Role of Targeted Therapy

tRNAs and tsRNAs, as small molecular markers related to tumor, may also play a role in molecular targeted therapy to suggest local therapeutic targets. For example, mt-tRNA mutations in lung cancer inhibit the development of NSCLC by leucyl-tRNA synthase mediated rapamycin complex 1 as a non-classical mammalian target (Kim et al., 2019). In high-grade serous ovarian cancer, tRF-03357 promotes cell proliferation, migration and invasion, partly by modulating HMBOX1. And this phenomenon can be reversed by targeting tRF-03357 (Zhang et al., 2019a). These functions indicate that tRNAs and tsRNAs can be used as therapeutic targets for clinical interventions. However, as a therapeutic target, more precise mechanisms of action and higher sensitivity specificity are required, which still leaves some unexplored areas.

## Conclusion and Perspectives

Recent studies have revealed they are key role to various human diseases’ development, especially cancer. Meanwhile, tsRNAs are increasingly being tapped for its potential role in diseases. This review summarizes recent literature on the biogenesis, structure, and biological characteristics and functions of tRNAs and tsRNAs, with an extraordinary attention to their participation and potential clinical significance in human cancers.

In human cancer, tRNAs and tsRNAs possess carcinogenic roles by promoting cell proliferation, migration, and invasion and inhibiting apoptosis (Santos et al., 2019). Meanwhile, tRNAs and tsRNAs also have anti-tumor effects. Although in clinical settings, fluid screening mainly focuses on miRNAs (Hayes et al., 2014; Romaine et al., 2015). However, tRNAs and tsRNAs are stably enriched in the biofluids in solid cancers and blood malignancies, (Zhang et al., 2009; Li et al., 2012), fluid screening can determine tRNAs and tsRNAs biomarker candidates for cancer diagnosis.

Several studies have confirmed that tRNAs and tsRNAs regulate cancer progression. Indeed, such mechanisms may become suitable targets for novel therapeutic approaches in several tumor types. Although tRNA has already been confirmed as a regulator, it remains unclear whether its dysregulation in many cancers is a trigger for tumor initiation, progress, or metastasis.

Compared with that on other non-coding RNAs, i.e., miRNA and lncRNA, the current state of knowledge on tRNAs and tsRNAs are still in its infancy, and they have not yet been studied in clinical settings. There are several challenges and limitations in the study of tRNAs. First, the molecular mechanism of tRNAs and tsRNAs in cancer development needs to be further confirmed. Second, most studies on tRNAs and tsRNAs and their role in cancer used cancer cells. To advance research in this field, future studies should utilize clinical samples, such as tumor tissues and body fluids. Third, how to utilize tRNAs and tsRNAs against cancer cells effectively and with long-term efficacy should be sufficiently addressed. Fourth, to ensure their safety and efficacy in human, pre-clinical and clinical studies are warranted. Lastly, the relationship between mutations in mitochondrial tRNA and maternal genetic diseases needs to be more clearly studied.

In conclusion, this review bridges the gap between what is known about tRNAs and tsRNAs and their involvement in human cancer, thus providing new insights and strategies for cancer diagnosis, management, and treatment (Hopper and Phizicky, 2003).

## Abbreviations

KLF, Kruppel-like factor; nts, nucleotides; pre-tRNAs, precursor tRNAs; tRFs, tRNA-derived fragments; tRNAs, transfer RNAs; tsRNAs, tRNA-derived small RNAs; tiRNAs, tRNA-derived stress-induced RNAs; YBX-1, Y-box binding protein 1.
